# The Impact of Social Isolation Due to COVID-19 on Symptom Progression in People With Dementia: Findings of the SOLITUDE Study

**DOI:** 10.3389/fpsyt.2022.877595

**Published:** 2022-05-10

**Authors:** Riccardo Manca, Matteo De Marco, Amanda Colston, Vanessa Raymont, Jay Amin, Rhys Davies, Pramod Kumar, Gregor Russell, Daniel J. Blackburn, Annalena Venneri

**Affiliations:** ^1^Department of Life Sciences, Brunel University London, Uxbridge, United Kingdom; ^2^Oxford Health NHS Foundation Trust, Oxford, United Kingdom; ^3^Department of Psychiatry, University of Oxford, Oxford, United Kingdom; ^4^Clinical Neurosciences, Clinical and Experimental Sciences, Faculty of Medicine, University of Southampton, Southampton, United Kingdom; ^5^Memory Assessment and Research Centre, Moorgreen Hospital, Southern Health NHS Foundation Trust, Southampton, United Kingdom; ^6^The Walton Centre NHS Foundation Trust, Liverpool, United Kingdom; ^7^Berkshire Healthcare NHS Foundation Trust, Bracknell, United Kingdom; ^8^Bradford District Care NHS Foundation Trust, Bradford, United Kingdom; ^9^Department of Neuroscience, University of Sheffield, Sheffield, United Kingdom

**Keywords:** dementia, COVID-19, social isolation, neuropsychiatric symptoms, cognitive decline

## Abstract

**Background:**

People with dementia (PWD) are vulnerable to abrupt changes to daily routines. The lockdown enforced on the 23rd of March 2020 in the UK to contain the expansion of the COVID-19 pandemic limited opportunities for PWD to access healthcare services and socialise. The SOLITUDE study explored the potential long-term effects of lockdown on PWD’s symptoms and carers’ burden.

**Methods:**

Forty-five carers and 36 PWD completed a telephone-based assessment at recruitment (T0) and after 3 (T1) and 6 months (T2). PWD completed measures validated for telephonic evaluations of cognition and depression. Carers completed questionnaires on their burden and on PWD’s health and answered a customised interview on symptom changes observed in the initial months of lockdown. Longitudinal changes were investigated for all outcome variables with repeated-measures models. Additional *post hoc* multiple regression analyses were carried out to investigate whether several objective factors (i.e., demographics and time under social restrictions) and carer-reported symptom changes observed following lockdown before T0 were associated with all outcomes at T0.

**Results:**

No significant changes were observed in any outcomes over the 6 months of observations. However, *post hoc* analyses showed that the length of social isolation before T0 was negatively correlated with episodic and semantic memory performance at T0. Carers reporting worsening of neuropsychiatric symptoms and faster disease progression in PWD also reported higher burden. Moreover, carer-reported worsening of cognitive symptoms was associated with poorer semantic memory at T0.

**Conclusion:**

PWD’s symptoms and carers’ burden remained stable over 6 months of observation. However, the amount of time spent under social restrictions before T0 appears to have had a significant detrimental impact on cognitive performance of patients. In fact, carer-reported cognitive decline during social isolation was consistent with the finding of poorer semantic memory, a domain sensitive to progression in Alzheimer’s disease. Therefore, the initial stricter period of social isolation had greater detrimental impact on patients and their carers, followed then by a plateau. Future interventions may be designed to maintain an optimal level of social and cognitive engagement for PWD in challenging times, to prevent abrupt worsening of symptoms and associated detrimental consequences on patients’ carers.

## Introduction

Quality of health and life expectancy are deeply influenced by the characteristics of our social environment. It has long been established that a series of quantitative and qualitative features of one’s social connections, and the social support that may derive from these, can variably but significantly affect several health domains, including cognitive health (1). Such detrimental effects appear to be particularly evident in the ageing population. Evans et al. (2) found that socially isolated older people with depression and/or anxiety show worse cognitive performance than those who are more socially connected. Both loneliness and social isolation have also been found to be associated with greater cognitive decline in older adults above 50 years of age, independently of depressive symptoms (3). Along these lines, several epidemiological studies and meta-analyses have consistently observed that smaller social networks (4), lack of close relationships (5), poor social engagement (6), loneliness and social isolation (7–9) are all associated with a higher risk of dementia. These findings suggest that an impoverished social environment can either foster or worsen cognitive decline in older adults both via a direct, e.g., lack of mental stimulation, and an indirect pathway, e.g., as a consequence of the impact on mental health.

In early 2020, strict limitations to social contacts were imposed in the United Kingdom to contain the Coronavirus Disease 2019 (COVID-19) pandemic. Although these campaigns have seen periods of strict restrictions (including lockdowns) alternating to phases of more relaxed regulations, people have been unable to carry out a normal and light-hearted social life for a prolonged period of time. This has brought unprecedented changes to daily-life conditions of people less accustomed to communication technology (e.g., older adults), and has resulted in a severe long-term reduction of light-hearted social life. Leaving aside all criticisms that have been raised by stakeholders on the adoption of social isolation measures (the discussion of which is not relevant to the aim of this paper), repeated and prolonged periods of lockdown have offered a unique opportunity for “natural experiments” that have enabled researchers to investigate, in an ecological setting, the impact of abruptly imposed social isolation on older people’s health. As expected, the detrimental effects of social restrictions on mental health and cognitive decline in older adults with or without cognitive impairments were observed early on during the COVID-19 pandemic (10). This impact may have been particularly severe in older people with selective risk factors, e.g., hearing loss (11), that may exacerbate isolation and, in turn, increase subjective perceptions of loneliness, and of decline in cognitive and mental health. Indeed, several observational studies carried out across the world have consistently detected worsening of existing and emergence of new neuropsychiatric symptoms in patients with dementia, after the introduction of a range of diverse measures of social isolation (12–16). As a possible consequence of the behavioural alterations experienced by people with dementia (PWD), negative effects were also reported on the burden and mental health of their carers (13, 17, 18).

In a similar fashion, the sudden and unforeseeable adoption of significant forms of restriction to social contacts may have fostered a worrying acceleration in the annual rates of cognitive decline in people with cognitive impairments compared with those observed in the years prior to the beginning of the COVID-19 pandemic (19, 20). Memory was found to be a particularly vulnerable cognitive domain (19). These results suggest that social restrictions may have created the ideal conditions for an acceleration of decline in PWD. This has been observed in a recent survey of 339 Greek carers of PWD: cognitive decline was reported in patients, especially in those with moderate-to-severe dementia, together with an increase in carers’ burden (21). Gan et al. (22) found signs of significant objective decline in several screening measures of global cognitive status, behavioural symptoms and daily-living activities in a sample of 205 older people with and without cognitive impairment assessed before and after enforcement of lockdown in China. A study that investigated the pre- vs. post-lockdown cognitive changes in patients with mild cognitive impairment and dementia due to Alzheimer’s disease found significant decline especially in verbal long-term memory and phonemic fluency (23).

These early findings support the claims that social isolation may be, indeed, detrimental to cognitive health in older adults, in general, and even more so in PWD. However, the impact that lockdown and quarantine measures may have had on specific cognitive domains and quality of life of patients with cognitive impairments and their potential long-lasting effects have not been clarified. Indeed, so far most investigations have only used screening measures for global cognitive decline (e.g., Mini Mental State Examination and Montreal Cognitive Assessment) and/or assessed patients’ cognitive performance only once, a few weeks after the introduction of social isolation measures. The SOcial LImitations Turn Up DEmentia (SOLITUDE) (24, 25) study was set up as a multi-centre observational longitudinal study to investigate these issues in the longer term, to document changes in cognitive performance, mental health and quality of life of PWD and to assess burden of their carers over 6 months since the first lockdown was enforced in the UK [for details of the full protocol see (26)].

## Materials and Methods

### Participants

Thirty-six PWD-carer dyads and 9 unaccompanied carers were recruited between September 2020 and March 2021 from 6 secondary-care neurology/old age psychiatry clinics in the UK. Inclusion criteria were: (1) a clinical diagnosis of dementia due to any neurodegenerative aetiology (mixed cases were included if the neurodegenerative condition was the main aetiology); (2) availability of a clinical assessment of global cognitive status with a score equivalent to a Mini Mental State Examination (MMSE) score ≥ 18 (for participants screened with a scale different from MMSE, the scores were converted to an equivalent MMSE score using available conversion tables).

PWD were excluded based on the following criteria: (1) major medical diagnoses other than dementia that could affect patient’s and carer’s physical and mental wellbeing; (2) non-neurodegenerative conditions as the primary cause of dementia; (3) history of long-term psychiatric conditions; (4) history of significant acute neurological events (e.g., stroke, traumatic brain injury); (4) absence of a reliable carer; (5) major sensory or speech impairments preventing telephone assessment; (6) no telephone service in place; (7) insufficient mastery of English. If an eligible PWD was not willing to participate, but his/her carer was, the sole carer was recruited. Exclusion criteria 5–7 were applied to the carer as well.

### Protocol of Assessments

All procedures were carried out in compliance with the Declaration of Helsinki. Ethical approval was granted by the NHS Health Research Authority, North West—Preston Regional Ethics Committee, reference n. 20/NW/0305 (protocol version 1).

The recruitment process, as already reported in a previous study (26), involved an initial screening of eligible candidates who were first contacted by a clinician and provided with the study’s information sheet. No longer than 1 week since receipt of the information material, all people (both PWD and carers) willing to take part in the study provided their audio-recorded informed consent over the telephone.

**FIGURE 1:**
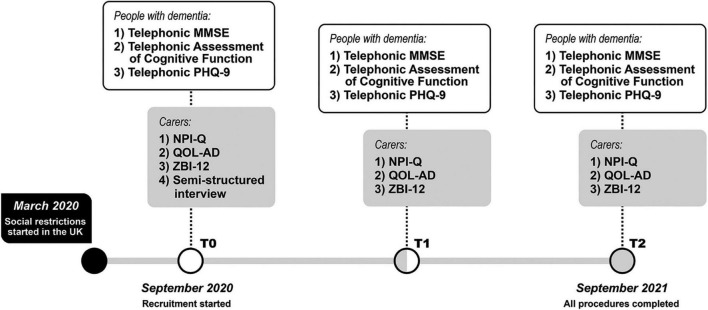
Timeline of the SOLITUDE study.

Participants underwent 3 telephone assessments: at recruitment (T0), at 3 months (T1) and at 6 months (T2) (see Figure 1 for a full timeline). The outcome variables collected during each assessment included cognitive tests validated for telephone administration and a series of questionnaires designed to be used with PWD and carers. Patients’ cognitive abilities were assessed using: the telephone Mini Mental State Examination (t-MMSE) (27) and the Telephone Assessment of Cognitive Function (28), i.e., a brief battery of tests comprising the Digit Span (forward and backward) and Digit Ordering tests, the Logical Memory test (immediate and delayed recall) and the Category Fluency test (animals and vegetables). Moreover, participants also completed the 9-item Patient Health Questionnaire validated for telephone assessment (29).

Outcome measures collected from carers were assessed by using 3 questionnaires validated for telephone assessments (30–32): the Neuropsychiatric Inventory Questionnaire (NPI-Q) (33) to evaluate PWD’s behavioural symptoms; the Quality of Life in Alzheimer’s Disease questionnaire (34) to provide information on several areas contributing to PWD’s quality of life; and the 12-item Zarit Burden Interview (ZBI-12) (35) to assess carer’s burden associated with caring for the PWD.

Moreover, only at T0, each carer completed a semi-structured interview adapted from one used in previous studies (15, 17). This interview included questions on patients, living conditions and socialisation before lockdown, carers’ personal mental health problems experienced and help received during lockdown, as well as carer-reported changes in PWD’s symptoms during lockdown (up to T0). Findings from the carer semi-structured interview have already been reported in Manca et al. (26). For the purpose of this study, only carer-reported changes in existing neuropsychiatric and cognitive symptoms, the emergence of new neuropsychiatric symptoms and carers’ concerns about progression of dementia were considered, among the variables collected as part of this customised interview, as predictors of all of the outcome measures.

### Statistical Analysis

First, all tests of the Telephone Assessment of Cognitive Function were z-transformed and used to calculate five composite indices at each time point: global cognition (average of all z-transformed tests), declarative memory (average of Logical Memory and Category Fluency z scores), episodic memory (average of Logical Memory z scores), semantic memory (average of Category Fluency z scores), and working memory (average of Digit Span and Digit Ordering z scores).

Longitudinal changes from T0 to T1, from T1 to T2 and from T0 to T2 were assessed for all outcome measures using repeated-measures ANCOVA models (the threshold of statistical significance was set to *p* = 0.05). The covariates included in the analyses were: patients’ age in years at T0, years of education, sex, last clinical MMSE score available before lockdown (as described in the section on inclusion criteria), time elapsed between last pre-lockdown MMSE and T0 assessment (in days) and time elapsed between the official beginning of lockdown in the United Kingdom (23rd March 2020) and the T0 assessment (in days). For variables pertaining to carers’ mental health, the carers’ years of age at T0, years of education and sex were included in the models as covariates.

Since the procedures of recruitment for the SOLITUDE study began 24 weeks after lockdown had been announced (this was to comply with completion of administrative requirements by the organisation sponsoring the study and obtain ethical approval), we decided to investigate whether the time spent under social restrictions enforced in the United Kingdom was associated with cognitive performance and wellbeing outcomes at T0. Therefore, several *post hoc* analyses were carried out additional to those planned *a priori* in the registered SOLITUDE study protocol: (1) a repeated-measures ANCOVA model to investigate changes in MMSE scores from pre-lockdown to T0, including the difference in time between the two assessments as a covariate; (2) multiple regression models to predict cognitive performance and wellbeing of both carers and PWD at T0 including the time elapsed between 23rd March 2020 and T0 assessment as predictor and the same covariates used in the repeated-measures models (i.e., age, education, pre-lockdown MMSE score, time elapsed between pre-lockdown MMSE and T0); (3) repetition of the same multiple regression models including also carer-reported changes in PWD’s symptoms (i.e., existing behavioural, cognitive, and motor, as well as new behavioural symptoms observed in the T0 semi-structured interview reported in Supplementary Table 1) as binary predictors (changes reported vs. no changes) to investigate the association between carers’ observation (covering the period of time between the enforcement of social isolation measures and T0) and objectively assessed outcome measures; (4) same regression models described in point (2) and point (3), but with the exclusion of pre-lockdown MMSE score from the covariate range, to predict changes in MMSE scores occurred before T0 captured by an MMSE difference score (pre-lockdown t-MMSE—T0 t-MMSE, calculated after converting the pre-lockdown MMSE to an equivalent t-MMSE score using conversion tables).

## Results

Demographic and clinical characteristics of all PWD and carers are reported in Table 1. The majority of patients received a clinical diagnosis of Alzheimer’s disease and the carer was their spouse/partner in most cases [for more details on our sample see (26)].

**TABLE 1:** Demographic characteristics of people with dementia and carers (mean ± *SD*).

Variable	All PWD (*n* = 45)	PWD directly assessed (*n* = 36)	Carers (*n* = 45)
Age (years)	74.04 ± 9.33	72.25 ± 8.55	69.24 ± 10.23
Education (years)	12.96 ± 3.01	13.25 ± 3.12	13.67 ± 2.99
Sex (M/F)	25/20	23/13	18/27
Pre-lockdown t-MMSE	20.93 ± 3.37	21.26 ± 3.37	–
**Diagnosis^a^:**			
*AD*	34 (75.6%)	28 (77.8%)	–
*Mixed aetiology*	5 (11.1%)	2 (5.6%)	–
*DLB*	3 (6.7%)	3 (8.3%)	–
*PCA*	2 (4.4%)	2 (5.6%)	–
*CBD*	1 (2.2%)	1 (2.7%)	–
**Relation with PWD^a^**			
*Spouse/partner*	–	–	38 (84.5%)
*Child*	–	–	6 (13.3%)
*Friend/acquaintance*	–	–	1 (2.2%)

*^a^Frequencies (proportions).*

*AD, Alzheimer’s disease; CBD, Corticobasal degeneration; DLB, Dementia with Lewy Bodies; PCA, Posterior cortical atrophy; PWD, People with dementia; t-MMSE, telephone Mini Mental State Examination.*

Of the 36 PWD who agreed to take part and completed study procedures at T0, only 32 completed the full assessment at T1 (1 patient completed only the t-MMSE at this time point) and 29 (80.5%) completed the full study (Table 2). Forty-five carers were recruited and, of these, 36 (80%) completed all assessments. Frequencies of carer-reported changes in patients’ symptoms over the first months spent under social restrictions are summarised in Supplementary Table 2.

**TABLE 2:** Changes in cognitive and clinical variables over the 6 months of observation.

Variable	T0-T1 change		T1-T2 change		T0-T2 change	
PWD—cognitive battery	*F* ^a^	*p*	*F* ^a^	*p*	*F* ^a^	*p*
t-MMSE	0.12	0.73	2.70	0.11	3.11	0.09
DSF	0.13	0.72	0.86	0.36	1.90	0.18
DSB	0.19	0.77	0.86	0.36	0.15	0.70
DO	0.08	0.78	1.39	0.25	0.01	0.91
LM—IR	0.19	0.77	0.06	0.81	0.07	0.80
LM—DR	0.37	0.55	0.68	0.42	0.04	0.83
CFa—total	1.64	0.21	3.15	0.09	0.70	0.41
CFv—total	0.09	0.76	0.83	0.37	0.11	0.74
CFa—I	0.73	0.40	0.10	0.76	0.11	0.74
CFa—P	0.12	0.73	0.03	0.87	0.10	0.75
CFv—I	0.02	0.89	0.02	0.89	0.03	0.87
CFv—P	0.10	0.76	0.48	0.49	0.12	0.73
**PWD**—**composite indices**						
GC-CI	0.07	0.79	1.12	0.30	1.03	0.32
WM-CI	0.08	0.78	0.06	0.82	0.34	0.56
DM-CI	0.54	0.47	0.42	0.52	0.41	0.53
EM-CI	0.01	0.93	0.04	0.85	0.00	0.96
SM-CI	1.04	0.32	**5.34**	**0.03**	0.89	0.36
**PWD**—**mental health**						
PHQ-9	0.89	0.35	0.58	0.45	1.50	0.23
**Carer-reported**						
QoL-AD	0.47	0.50	0.03	0.85	0.04	0.83
NPI-Q—total	0.67	0.42	0.07	0.79	0.06	0.82
NPI-Q—distress	2.52	0.12	0.06	0.81	0.01	0.93
ZBI-12	0.38	0.54	0.12	0.73	2.86	0.10

*^a^F-statistic associated with the variable “Time” in repeated-measures models.*

*CFa/CFv, Category Fluency test–animals/vegetables (I, Intrusions; P, Perseverations); DM-CI, Declarative Memory Composite Index; DO, Digit Ordering test; DSB, Digit Span test—backward; DSF, Digit Span test–forward; EM-CI, Episodic Memory Composite Index; GC-CI, Global Cognitive Composite Index; LM, Logical Memory test (DR: Delayed recall, IR: Immediate recall); NPI-Q, Neuropsychiatric Inventory Questionnaire; PHQ-9, 9-item Patient Health Questionnaire; PWD, People with dementia; QoL-AD, Alzheimer’s Disease Quality of Life; SM-CI, Semantic Memory Composite Index; t-MMSE, telephone Mini Mental State Examination; WM-CI, Working Memory Composite Index; ZBI-12, 12-item Zarit Burden Interview. All significant results are reported in bold.*

Repeated-measures ANCOVA models revealed no changes in any of the outcome measures between any time points, apart from a weak improvement on the semantic memory composite index between T1 and T2 (*F* = 5.34, *p* = 0.03) (Table 2; see Supplementary Table 3 for full descriptive statistics).

*Post hoc* analyses showed no significant changes in t-MMSE scores from before lockdown (*F* = 0.013, *p* = 0.91). However, multiple regression analyses revealed that the time spent under social restrictions before T0 was negatively associated with cognitive performance of PWD on the Logical Memory test, both immediate (β = −0.39, *p* = 0.03, r^2^_part_ = 0.11) and delayed recall (β = −0.46, *p* < 0.01, r^2^_part_ = 0.16), and with scores on the Category Fluency test—animals (β = −0.44, *p* < 0.01, r^2^_part_ = 0.14) (Table 3). Similarly, a negative association was also detected with all composite indices, apart from the working memory composite index, with small-to-medium effect size (36) (global cognition: r^2^_part_ = 0.14, declarative memory: r^2^_part_ = 0.18, episodic memory: r^2^_part_ = 0.15, semantic memory: r^2^_part_ = 0.13). Lower pre-lockdown MMSE score was significantly associated with worse global cognitive and episodic memory performance. Higher levels of education significantly predicted higher scores on most cognitive tests. Moreover, both higher education and younger age were associated with less severe neuropsychiatric symptomatology (i.e., lower NPI-Q scores).

**TABLE 3:** Results of the multivariate multiple regression models (βs and standard errors) to predict cognitive and clinical characteristics of PWD and carers at T0.

T0 variables	Age (years)	Education (years)	Sex	Pre-lockdown MMSE	Time of social restrictions (days)
**PWD**—**cognitive battery**					
t-MMSE	0.03 (0.07), *p* = 0.87	**0.32 (0.17), *p* = 0.03**	0.09 (1.13), *p* = 0.53	**0.44 (0.17), *p* < 0.01**	−0.27 (0.01), *p* = 0.11
DSF	0.22 (0.03), *p* = 0.25	**0.51 (0.07), *p* < 0.01**	−0.11 (0.46), *p* = 0.50	−0.20 (0.07), *p* = 0.26	−0.04 (0.01), *p* = 0.85
DSB	0.24 (0.04), *p* = 0.22	−0.03 (0.08), *p* = 0.87	−0.21 (0.50), *p* = 0.26	0.36 (0.07), *p* = 0.06	−0.12 (0.01), *p* = 0.54
DO	0.09 (0.02), *p* = 0.59	**0.37 (0.07), *p* = 0.02**	0.26 (0.47), *p* = 0.08	0.29 (0.07), *p* = 0.07	−0.13 (0.01), *p* = 0.41
LM—IR	0.03 (0.01), *p* = 0.87	0.17 (0.23), *p* = 0.29	−0.02 (1.48), *p* = 0.88	**0.50 (0.22), *p* < 0.01**	−**0.39 (0.01), *p* = 0.03**
LM—DR	0.02 (0.01), *p* = 0.91	0.14 (0.31), *p* = 0.35	−0.14 (2.06), *p* = 0.38	**0.51 (0.31), *p* < 0.01**	−**0.46 (0.02), *p* < 0.01**
CFa—total	0.01 (0.01), *p* = 0.97	**0.41 (0.19), *p* = 0.01**	0.16 (1.22), *p* = 0.30	0.11 (0.18), *p* = 0.47	−**0.44 (0.01), *p* = 0.01**
CFv—total	−0.16 (0.08), *p* = 0.39	0.22 (0.20), *p* = 0.20	−0.03 (1.30), *p* = 0.84	0.27 (0.19), *p* = 0.14	−0.32 (0.01), *p* = 0.09
CFa—I	−0.04 (0.01), *p* = 0.86	0.13 (0.01), *p* = 0.49	−0.07 (0.09), *p* = 0.71	−0.09 (0.01), *p* = 0.66	−0.12 (0.01), *p* = 0.59
CFa—P	0.18 (0.03), *p* = 0.35	−0.09 (0.09), *p* = 0.63	−0.21 (0.57), *p* = 0.25	0.04 (0.08), *p* = 0.82	0.19 (0.01), *p* = 0.34
CFv—I	**0.36 (0.02), *p* = 0.05**	−0.05 (0.05), *p* = 0.73	0.27 (0.31), *p* = 0.10	−0.19 (0.05), *p* = 0.28	−0.31 (0.01), *p* = 0.09
CFv—P	−0.02 (0.03), *p* = 0.93	−0.06 (0.07), *p* = 0.73	−0.34 (0.42), *p* = 0.07	−0.01 (0.06), *p* = 0.98	0.05 (0.01), *p* = 0.81
**PWD**—**composite indices**					
GC-CI	0.10 (0.01), *p* = 0.52	**0.40 (0.03), *p* < 0.01**	−0.02 (0.19), *p* = 0.88	**0.42 (0.03), *p* < 0.01**	−**0.43 (0.01), *p* = 0.01**
WM-CI	0.28 (0.01), *p* = 0.12	**0.43 (0.03), *p* = 0.01**	−0.03 (0.21), *p* = 0.87	0.23 (0.03), *p* = 0.19	−0.15 (0.01), *p* = 0.41
DM-CI	−0.03 (0.02), *p* = 0.87	0.28 (0.04), *p* = 0.05	−0.01 (0.24), *p* = 0.94	**0.43 (0.04), *p* < 0.01**	−**0.49 (0.01), *p* < 0.01**
EM-CI	0.03 (0.02), *p* = 0.87	0.17 (0.04), *p* = 0.26	−0.09 (0.28), *p* = 0.56	**0.54 (0.04), *p* < 0.01**	−**0.46 (0.01), *p* < 0.01**
SM-CI	−0.85 (0.02), *p* = 0.62	**0.34 (0.04), *p* = 0.03**	0.07 (0.29), *p* = 0.67	0.21 (0.04), *p* = 0.20	−**0.42 (0.01), *p* = 0.02**
**PWD—mental health**					
PHQ-9	−0.34 (0.09), *p* = 0.08	−0.21 (0.22), *p* = 0.23	0.03 (1.47), *p* = 0.88	0.31 (0.22), *p* = 0.10	0.15 (0.01), *p* = 0.44
**Carer-reported**					
QOL-AD	−0.09 (0.15), *p* = 0.64	0.26 (0.36), *p* = 0.13	−0.19 (2.39), *p* = 0.27	−0.06 (0.36), *p* = 0.73	−0.12 (0.02), *p* = 0.52
NPI-Q—total	−**0.41 (0.11), *p* = 0.03**	−**0.39 (0.27), *p* = 0.02**	0.13 (1.79), *p* = 0.43	0.27 (0.27), *p* = 0.14	0.26 (0.02), *p* = 0.17
NPI-Q—distress	−0.30 (0.13), *p* = 0.09	−0.09 (0.43), *p* = 0.59	−0.08 (2.46), *p* = 0.62	−0.02 (0.37), *p* = 0.89	0.32 (0.02), *p* = 0.05
ZBI-12	−0.07 (0.16), *p* = 0.71	−0.08 (0.55), *p* = 0.65	0.15 (3.15), *p* = 0.38	−0.08 (0.48), *p* = 0.63	0.06 (0.02), *p* = 0.37

*CFa/CFv, Category Fluency test—animals/vegetables (I, Intrusions; P, Perseverations); DM-CI, Declarative Memory Composite Index; DO, Digit Ordering test; DSB, Digit Span test—backward; DSF, Digit Span test—forward; EM-CI, Episodic Memory Composite Index; GC-CI, Global Cognitive Composite Index; LM, Logical Memory test (DR, Delayed recall; IR, Immediate recall); NPI-Q, Neuropsychiatric Inventory Questionnaire; PHQ-9, 9-item Patient Health Questionnaire; PWD, People with dementia; QoL-AD, Alzheimer’s Disease Quality of Life; SM-CI, Semantic Memory Composite Index; t-MMSE, telephone Mini Mental State Examination; WM-CI, Working Memory Composite Index; ZBI-12, 12-item Zarit Burden Interview. All significant results are reported in bold.*

**FIGURE 2:**
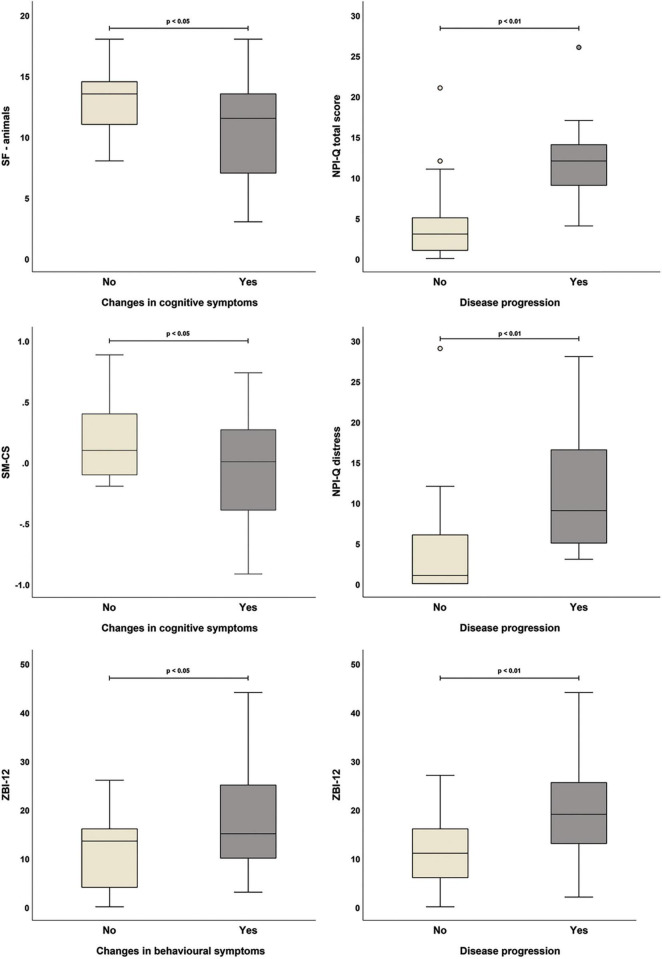
Significant associations between carer-reported changes in patients’ symptoms and outcome measures collected at T0 (all variables were treated as binary: yes, symptom changes/faster progression reported by carer; no, carer reported no symptom changes/faster progression).

Carer-reported cognitive decline was associated with worse performance on the Category Fluency test (“animals” category) and with lower semantic memory composite indices at T0 (Figure 1; see Supplementary Table 4). Carers’ impression of faster disease progression was associated with higher NPI-Q scores and worse carers’ distress and burden. Moreover, worsening of behavioural symptoms observed by carers was also significantly associated with higher carer-reported burden (i.e., higher ZBI-12 scores) (Figure 2).

Finally, no significant associations were detected between any of the objective and subjective (i.e., carer-reported) factors investigated and the MMSE difference score (Supplementary Table 5).

## Discussion

Our sample of PWD primarily due to neurodegenerative aetiologies had been cognitively and behaviourally stable over the 6-month timeframe of the SOLITUDE study, despite their adherence to the rules imposing restrictions to social contacts. Similarly, no significant changes were observed in the levels of carers’ distress and burden. This period of observation, however, occurred at a time when people had already been experiencing restrictions to their social routines for several months. This might have given them the opportunity to develop a degree of adjustment and might have prompted them to make targeted adaptations to cope with the practical consequences of enforced social limitations. Investigations into the factors that might have been associated with the outcome measures assessed at T0 highlighted that the number of days spent under social restrictions was negatively associated with patients’ performance. This was particularly detectable on tests of episodic and semantic memory. Moreover, scores on the Category fluency test at T0 were found to be significantly lower in PWD who were judged by their carers to have worsened cognitively over the first months of lockdown than in those who had been said to have remained stable. Carers who thought that the PWD experienced symptom worsening, both behaviourally and/or in association with their general clinical profile, also reported significantly higher burden and distress scores than carers who noticed no changes.

The findings of the SOLITUDE study are in line with those of similar recent studies and seem to suggest lockdown-related decline in some cognitive domains, i.e., semantic fluency and long-term memory, in patients with cognitive impairment due to AD (23) and even other types of neurodegenerative conditions (19). In fact, the duration of the period of forced social isolation was negatively associated with patients’ memory performance at T0. On the contrary, no significant general decline was detected by means of the t-MMSE in the same timeframe, and changes on this scale were associated neither with the time spent under social restrictions nor with the carer-reported changes in patients’ symptoms. This suggests that a sudden reduction in social stimulation that is protracted over a long period of time may exert detrimental effects on specific cognitive abilities in PWD, as also found by a longitudinal study that followed up patients with AD and Lewy Body dementia over 1 year (37). These specific declines are not captured if simple screening instruments like the MMSE are used and may go undetected if assessment of cognitive status of PWD is limited to global staging measures, especially in patients with a mild level of severity. A mildly significant improvement of the semantic memory composite index was, however, noted from T1 to T2. This finding could be due either to practice effect, since the same two semantic categories were used for all assessments, or to random variation in performance, since a non-significant trend toward a decline in this composite index was noted from T0 to T1. It must be noted that some degree of practice effect may possibly explain also the lack of decline over the 6-month time frame of this study in all cognitive domains assessed.

It is possible that protracted social isolation may have had a direct impact on cognitive health of PWD by limiting the opportunities either to practice their cognitive skills and strategies that were still preserved before the enforcement of lockdown or to acquire new strategies to cope with cognitive decline, i.e., cognitive reserve of patients may have been depleted by lack of social stimulation (38). The importance of cognitive reserve is suggested by the significant associations found between education and clinical profiles at T0, i.e., better performance on most cognitive tests and lower NPI-Q scores. Although we found no significant changes in PWD’s neuropsychiatric symptoms, either patient- or carer-reported, it is also likely that socially isolated patients may experience more severe behavioural and psychological symptoms (12–16) that may precipitate cognitive decline (39, 40). Indeed, social networks can provide support for patients resulting in better physical and mental health (41). These detrimental effects could explain the epidemiological link between social isolation and increased risk of dementia (42), and greater levels of AD-related neural damage, as highlighted by human neuropathological studies (43). Moreover, a few recent studies investigated experimentally the neural mechanisms that could underpin this association and found that social isolation seems to foster AD pathology accumulation in an animal model of this disease (43).

To the best of our knowledge, no PWD and carers were infected by SARS-CoV-2 either prior to or during participation in the SOLITUDE study, although we cannot fully rule out possible cognitive and/or behavioural disturbances that might have been caused by asymptomatic SARS-CoV-2 infections. Indeed, COVID-19 has been shown to cause neural damage and lead to cognitive decline (44), but this seems to be the case particularly in older people severely affected by the infection (45).

Levels of carers’ burden and distress caused by neuropsychiatric symptoms of PWD were also found to be stable over the observation period and no association was detected between these carer-related outcomes and any of the objective factors investigated. However, carer-reported worsening in the neuropsychiatric symptoms of PWD and faster disease progression over the first months of lockdown were significantly associated with higher burden and distress scores. Although we cannot exclude that carers’ mental health status might have influenced subjective perception of burden and distress (46), it must be noted that very similar findings emerged from other investigations into the consequences of measures of social restrictions enforcement due to the COVID-19 pandemic (13, 17, 18).

Interesting results emerged from the association between carer-reported cognitive decline and objectively assessed patients’ neuropsychological performance at T0. In fact, carer-reported worsening of cognitive symptoms just after lockdown (until recruitment) was negatively associated with the Category Fluency score (number of animals) and the semantic memory composite index. Therefore, carers’ judgments of cognitive health of PWD appeared to be in agreement with the objective observation of lower performance in semantic memory, a domain negatively affected by the amount of time spent in social isolation and that is sensitive to disease progression in AD (47). A recent cross-sectional study has also found greater cognitive and behavioural decline in PWD who were reported by their carers as more cognitively impaired since enforcement of social isolation regulations (48). This means that carers of PWD can provide clinically meaningful information on patients and this may be particularly helpful to clinicians when a direct assessment of the patient is not possible. Indeed, previous research has highlighted that carers can detect cognitive impairment accurately, although their assessment may not help differentiate different cognitive profiles (49, 50).

A first limitation of this study is the small sample size that, combined with a small number of drop-outs, might have prevented the detection of subgroups characterised by distinct patterns of longitudinal changes. However, despite the limited number of patients recruited, the association between the time spent under social restrictions and cognitive performance at T0 emerged as a significant finding [although with small and medium effect sizes, conventionally defined for multiple regression as effects in the range of 0.05–0.15 and of 0.15–0.35, respectively (36)]. As a consequence of the unforeseen circumstances that affected the great majority of the population, a control group of PWD who were not socially isolated could not be included. This prevents definite conclusions on the extent to which social isolation may have affected cognition in PWD. Second, our sample lacked patients from ethnic minorities, possibly due to a range of cultural (e.g., use of health services, interpretation of cognitive symptoms) and biological factors [e.g., higher rates of vascular cognitive impairment among certain ethnic minority groups, such as South Asians (51)]. Lack of evidence from ethnic minority groups, therefore, limits the generalisation of our conclusions to the whole clinical population of PWD due to neurodegenerative conditions, although it is highly likely that similar detrimental effects would be seen across populations of any ethno-racial background. Future studies are needed to clarify this pressing issue, considering that in the United Kingdom and other western countries, ethnic minorities have been affected by the COVID-19 pandemic more than White people (52). Third, the very small number of patients with non-AD dementias recruited for this study hindered any possibility of stratifying our sample by aetiology to gather insights into the differential impact of social isolation on people affected by different types of neurodegenerative diseases. Fourth, most carers were spouses/partners of PWD and this limited any possibility to analyse differences in outcome measures of burden between groups of carers differentially related to the PWD. Finally, it must be noted that the SOLITUDE protocol included no visuo-spatial, executive and social cognitive tests, primarily because of two reasons: (1) the nature of the assessment, i.e., telephone-based, that prevents the administration of visual stimuli, and (2) the lack of measures validated for remote research settings. Future efforts to develop tasks that could be delivered either via telephone or video-conference to assess a broader range of cognitive abilities in PWD will be beneficial to move the field of tele-neuropsychology forward.

Lockdown enforced to limit the current COVID-19 pandemic has extensively impacted everybody’s life, but also offered the conditions to study the impact of social isolation on cognitive health. The SOLITUDE study, consistently with other thematically aligned investigations world-wide, provides some insights indicating that a long-lasting reduction in social connectedness has an impact on objectively assessed cognitive performance of PWD, especially on semantic abilities. This finding was also supported by the consistent information provided by carers about changes in cognitive symptoms. Further studies in larger cohorts should ascertain what factors may either worsen or protect against the negative influence of social isolation on cognitive health of PWD. Moreover, investigations of interventions with the potential to limit cognitive decline resulting from either a reduction or lack in social connections for PWD are needed to devise and provide evidence-based support during challenging times like those caused by the COVID-19 pandemic (53).

## Data Availability Statement

The original contributions presented in the study are included in the article/Supplementary Material, further inquiries can be directed to the corresponding author/s.

## Ethics Statement

The studies involving human participants were reviewed and approved by NHS Health Research Authority, North West—Preston Regional Ethics Committee, reference n. 20/NW/0305 (protocol version 1). The patients/participants provided their written informed consent to participate in this study.

## Author Contributions

RM contributed to the study inception and participant recruitment, collected (part of), analysed and interpreted the data, drafted, revised, and approved the final version of the manuscript for submission. MDM conceived the study design, contributed to data interpretation, revised, and approved the manuscript for submission. AC, VR, JA, RD, PK, GR, and DJB led site-specific recruitment and data collection, revised, and approved the manuscript for submission. AV conceived the study, contributed to recruitment and data interpretation, revised, and finalised the manuscript for submission. All authors contributed to the article and approved the submitted version.

## Conflict of Interest

The authors declare that the research was conducted in the absence of any commercial or financial relationships that could be construed as a potential conflict of interest.

## Publisher’s Note

All claims expressed in this article are solely those of the authors and do not necessarily represent those of their affiliated organizations, or those of the publisher, the editors and the reviewers. Any product that may be evaluated in this article, or claim that may be made by its manufacturer, is not guaranteed or endorsed by the publisher.
