# Supplementary material for: The Impact of Social Isolation Due to COVID-19 on Symptom Progression in People With Dementia: Findings of the SOLITUDE Study
Source: Front Psychiatry. 2022 May 10;13:877595. doi: 10.3389/fpsyt.2022.877595 (PMC9127264; doi:10.3389/fpsyt.2022.877595)
Supplement: Supplementary file 1 [file Data_Sheet_1.docx]

Supplementary Material

# Supplementary Table 1. List of questions about changes in patients’ behavioural and cognitive symptoms from the interview to carers at T0 used for analyses in this study.

| **Question** | **Answers** |
| --- | --- |
| *1) Have you noticed any changes in the behaviour of the person with dementia since the introduction of social isolation measures?* | Yes/No |
| - If you answered yes to the previous question, what behavioural symptoms already present before the introduction of social isolation became more severe? | - Apathy - Anxiety - Depression - Sleep problems - Delusions - Hallucinations - Irritability - Aggression - Wandering - Agitation - Changes in appetite |
| *2) Have you noticed any new behavioural symptoms NOT present before the introduction of social isolation?* | - No new symptoms - Apathy - Anxiety - Depression - Sleep problems - Delusions - Hallucinations - Irritability - Aggression - Wandering - Agitation - Changes in appetite |
| *3) Have you noticed any changes in the cognitive symptoms of the person with dementia already present before the introduction of social isolation?* | - No - Yes, he/she is more confused - Yes, he/she is more forgetful - Yes, he/she has more difficulties in finding the right words - Yes, he/she is more disorientated at home and does not recognise it - Yes, he/she is more disorientated in time - Yes, he/she has more difficulties in recognising his/her own relatives - Yes, he/she has more difficulties in recognising himself/herself at the mirror |
| *4) Do you think that the disease has progressed more quickly during this period of social isolation?* | Yes/No |

Answers to all questions were treated as binary predictors (no change vs change) in *post hoc* multiple regression analyses.

**Supplementary Table 2.** Frequencies of carer-reported changes in symptoms in PWD.

| **Carer-reported changes** | **All PWD (*n* = 45)** | | **PWD directly assessed (*n* = 36)** | |
| --- | --- | --- | --- | --- |
|  | **Yes** | **No** | **Yes** | **No** |
| Existing behavioural symptoms | 23 (48.9%) | 22 (51.1%) | 15 (41.7%) | 21 (58.3%) |
| New behavioural symptoms | 12 (26.7%) | 33 (73.3%) | 7 (19.4%) | 29 (80.6%) |
| Existing cognitive symptoms | 33 (73.3%) | 12 (26.7%) | 24 (66.7%) | 12 (33.3%) |
| Faster disease progression | 19 (42.2%) | 26 (57.8%) | 11 (30.6%) | 25 (69.4%) |

**Supplementary Table 3.** Descriptive statistics of the cognitive and clinical variables at all time points (mean ± SD).

| **Variable** | **T0** | **T1** | **T2** |
| --- | --- | --- | --- |
| ***PWD – cognitive battery*** | ***(n = 36)*** | ***(n = 32)*** | ***(n = 29)*** |
| t-MMSE | 20.97 ± 3.75 | 20.88 ± 3.46 (*n = 33^b^*) | 20.93 ± 4.31 |
| DSF | 6.03 ± 1.32 | 5.72 ± 1.55 | 5.37 ± 1.57 |
| DSB | 3.86 ± 1.33 | 4.16 ± 1.19 | 4.17 ± 1.63 |
| DO | 4.53 ± 1.56 | 4.88 ± 1.54 | 5.21 ± 1.84 |
| LM – IR | 6.22 ± 4.58 | 8.03 ± 4.75 | 7.52 ± 5.77 |
| LM – DR | 6.44 ± 6.57 | 7.28 ± 6.77 | 8.31 ± 7.76 |
| CFa – total | 11.33 ± 3.92 | 12.81 ± 4.67 | 11.14 ± 4.37 |
| CFv – total | 6.64 ± 3.64 | 7.81 ± 2.57 | 7.62 ± 3.97 |
| CFa – I | 0.06 ± 0.23 | 0.06 ± 0.25 | 0.07 ± 0.37 |
| CFa – P | 1.22 ± 1.53 | 1.34 ± 1.70 | 2.07 ± 2.14 |
| CFv – I | 0.36 ± 0.93 | 0.13 ± 0.42 | 0.48 ± 0.83 |
| CFv – P | 0.75 ± 1.16 | 1.13 ± 1.29 | 0.76 ± 1.09 |
| ***PWD – composite indices*** |  |  |  |
| GC-CI | -0.03 (0.99)^a^ | 0.06 (0.89)^a^ | 0.18 (0.82)^a^ |
| WM-CI | 0.00 (1.07)^a^ | -0.06 (0.87)^a^ | 0.12 (0.82)^a^ |
| DM-CI | -0.12 (1.28)^a^ | -0.15 (0.82)^a^ | -0.05 (1.23)^a^ |
| EM-CI | -0.05 (1.52)^a^ | 0.17 (1.43)^a^ | 0.00 (1.98)^a^ |
| SM-CI | 0.13 (1.33)^a^ | -0.06 (1.28)^a^ | 0.03 (1.45)^a^ |
| ***PWD – mental health*** |  |  |  |
| PHQ-9 | 4.17 ± 4.09 | 3.94 ± 4.99 | 4.52 ± 4.82 |
| ***Carer-reported*** | ***(n = 45)*** | ***(n = 39)*** | ***(n = 36)*** |
| QoL-AD | 33.64 ± 7.33 | 32.56 ± 7.60 | 32.21 ± 6.71 |
| NPIQ – total | 7.44 ± 5.95 | 7.31 ± 7.05 | 7.86 ± 7.08 |
| NPIQ – distress | 7.27 ± 7.78 | 6.15 ± 6.99 | 6.75 ± 6.55 |
| ZBI-12 | 14.98 ± 9.53 | 12.77 ± 9.29 | 13.42 ± 8.72 |

^a^ Median (Interquartile range)

^b^ At T1, one patient decided to complete only the t-MMSE

CFa/CFv: Category Fluency test – animals/vegetables (I: Intrusions, P: Perseverations), DM-CI: Declarative Memory Composite Index, DO: Digit Ordering test, DSB: Digit Span test – backward, DSF: Digit Span test – forward, EM-CI: Episodic Memory Composite Index, GC-CI: Global Cognitive Composite Index, LM: Logical Memory test (DR: Delayed recall, IR: Immediate recall), NPIQ: Neuropsychiatric Inventory Questionnaire, PHQ-9: 9-item Patient Health Questionnaire, PWD: People with dementia, QoL-AD: Alzheimer’s Disease Quality of Life, SM-CI: Semantic Memory Composite Index, t-MMSE: telephone Mini Mental State Examination, WM-CI: Working Memory Composite Index, ZBI-12: 12-item Zarit Burden Interview

**Supplementary Table 4.** Multiple regression coefficients (βs) for carer-reported predictors of cognitive and clinical characteristics of PWD and carers at T0.

| **T0 variables** | **Changes in behavioural symptoms** | **New behavioural symptoms** | **Changes in cognitive symptoms** | **Perception of faster disease progression** |
| --- | --- | --- | --- | --- |
| ***PWD – cognitive battery*** | | | | |
| t-MMSE | 0.02, p = 0.91 | 0.14, p = 0.34 | -0.08, p = 0.59 | -0.13, p = 0.37 |
| DSF | -0.03, p = 0.86 | 0.13, p = 0.44 | 0.10, p = 0.55 | 0.30, p = 0.08 |
| DSB | -0.03, p = 0.86 | 0.33, p = 0.07 | 0.11, p = 0.56 | 0.17, p = 0.35 |
| DO | -0.05, p = 0.73 | -0.07, p = 0.62 | -0.23, p = 0.11 | -0.03, p = 0.82 |
| LM – IR | 0.23, p = 0.14 | 0.11, p = 0.51 | -0.11, p = 0.47 | -0.21, p = 0.19 |
| LM – DR | 0.02, p = 0.87 | 0.00, p = 0.98 | -0.04, p = 0.78 | -0.13, p = 0.40 |
| CFa – total | 0.11, p = 0.47 | 0.09, p = 0.55 | **-0.35, p = 0.02** | -0.21, p = 0.18 |
| CFv – total | 0.12, p = 0.48 | 0.14, p = 0.41 | -0.22, p = 0.20 | -0.23, p = 0.18 |
| CFa – I | 0.07, p = 0.72 | -0.13, p = 0.71 | 0.26, p = 0.19 | 0.21, p = 0.30 |
| CFa – P | -0.29, p = 0.09 | -0.28, p = 0.13 | -0.25, p = 0.16 | -0.12, p = 0.52 |
| CFv – I | 0.03, p = 0.85 | -0.08, p = 0.65 | 0.00, p = 0.99 | -0.17, p = 0.32 |
| CFv – P | -0.20, p = 0.26 | -0.23, p = 0.20 | -0.06, p = 0.76 | -0.15, p = 0.40 |
| ***PWD – composite indices*** | | | | |
| GC-CI | 0.08, p = 0.56 | 0.17, p = 0.25 | -0.17, p = 0.24 | -0.08, p = 0.59 |
| WM-CI | -0.06, p = 0.72 | 0.20, p = 0.23 | -0.01, p = 0.95 | 0.22, p = 0.18 |
| DM-CI | 0.15, p = 0.30 | 0.11, p = 0.47 | -0.22, p = 0.12 | -0.24, p = 0.10 |
| EM-CI | 0.14, p = 0.35 | 0.06, p = 0.70 | -0.08, p = 0.57 | -0.19, p = 0.21 |
| SM-CI | 0.13, p = 0.41 | 0.13, p = 0.42 | **-0.32, p = 0.04** | -0.24, p = 0.13 |
| ***PWD – mental health*** | | | | |
| PHQ-9 | -0.16, p = 0.36 | 0.15, p = 0.42 | 0.21, p = 0.22 | 0.07, p = 0.68 |
| ***Carer-reported*** |  |  |  |  |
| QOL-AD | -0.29, p = 0.07 | -0.01, p = 0.94 | -0.05, p = 0.76 | -0.07, p = 0.68 |
| NPIQ – total | 0.23, p = 0.16 | 0.01, p = 0.96 | 0.28, p = 0.09 | **0.47, p < 0.01** |
| NPIQ – distress | 0.19, p = 0.25 | 0.12, p = 0.47 | 0.23, p = 0.15 | **0.45, p < 0.01** |
| ZBI-12 | **0.35, p = 0.03** | 0.09, p = 0.58 | 0.29, p = 0.08 | **0.55, p < 0.01** |

AD-QOL: Alzheimer’s Disease Quality of Life, CFa/CFv: Category Fluency test – animals/vegetables (I: Intrusions, P: Perseverations), DM-CI: Declarative Memory Composite Index, DO: Digit Ordering test, DSB: Digit Span test – backward, DSF: Digit Span test – forward, EM-CI: Episodic Memory Composite Index, GC-CI: Global Cognitive Composite Index, LM: Logical Memory test (DR: Delayed recall, IR: Immediate recall), NPIQ: Neuropsychiatric Inventory Questionnaire, PHQ-9: 9-item Patient Health Questionnaire, PWD: People with dementia, SM-CI: Semantic Memory Composite Index, t-MMSE: telephone Mini Mental State Examination, WM-CI: Working Memory Composite Index, ZBI-12: 12-item Zarit Burden Interview

**Supplementary Table 5.** Multiple regression coefficients (βs) for objective and carer-reported predictors of changes in MMSE scores (pre-lockdown t-MMSE – T0 t-MMSE).

|  |  |  | ***Objective*** |  |  |
| --- | --- | --- | --- | --- | --- |
|  | **Age** | **Education** | **Sex** | **Time of social restrictions (days)** | **Time between assessments (days)** |
| MMSE difference | 0.06, p = 0.29 | -0.26, p = 0.13 | -0.01, p = 0.97 | 0.33, p = 0.09 | 0.14, p = 0.44 |
|  |  |  | ***Carer-reported*** |  |  |
|  | **Changes in behavioural symptoms** | **New behavioural symptoms** | **Changes in cognitive symptoms** | **Perception of faster disease progression** | --- |
| MMSE difference | 0.03, p = 0.88 | -0.12, p = 0.51 | 0.16, p = 0.35 | 0.25, p = 0.15 | **---** |

MMSE: Mini Mental State Examination
